# Toward Formal Models and Languages for Verifiable Multi-Robot Systems

**DOI:** 10.3389/frobt.2018.00094

**Published:** 2018-09-05

**Authors:** Rocco De Nicola, Luca Di Stefano, Omar Inverso

**Affiliations:** ^1^IMT School for Advanced Studies, Lucca, Italy; ^2^Gran Sasso Science Institute, L'Aquila, Italy

**Keywords:** multi-robot systems, languages, communication, collective behavior, automated reasoning

## Abstract

Incorrect operation of a multi-robot system (MRS) may not only lead to unsatisfactory results, but it can also cause economic losses and jeopardize safety. These risks may not always be evident, since they may arise as unforeseen consequences of interactions between different components of the system. Thus, tools and techniques that can help in providing guarantees about the behavior of MRSs are on demand; whenever possible, these guarantees should be backed up by formal proofs complementing the traditional approaches based on testing and simulation. Tailored linguistic support to specify MRSs is a major step toward this goal. In fact, reducing the gap between typical features of an MRS and the linguistic primitives used to model them would simplify both the specification of these systems and their verification. With the aim of reducing this gap, we identified some key features of MRSs in this work. Subsequently, we considered the selection of three specification languages oriented toward MRSs, which are representative of wider categories of languages with similar aims. Finally, we assessed the extent to which the considered languages captured the key features in an adequate and intuitive way by using them to implement multi-robot systems case studies.

## 1. Introduction

Multi-robot systems (MRSs) are an increasingly popular topic in robotics research. Their broad range of activities have been categorized in several different ways (Arai et al., 2002; Brambilla et al., 2013; Bayındır, 2016). Typical tasks of MRSs include exploration or patrolling, object transportation and manipulation (e.g., foraging), deployment (e.g., pattern formation), collective decision making (e.g., flocking), task allocation, and many others.

Cooperation is the real power of an MRS; by working together, the robots can globally achieve goals that would be “difficult, if not impossible, to be accomplished by an individual robot” (Arai et al., 2002). On the other hand, the concerns that typically arise with any robotic system (Vasic and Billard, 2013) are largely exacerbated by the presence of multiple cooperating units, whose incorrect operations can lead to economic losses and even threats to safety. Such concerns are related to the inherent features of MRSs rather than the specific kind of task of an MRS.

The first source of complexity is the *open-endedness* of MRSs, that is, the fact that robots can dynamically enter or leave the system. This happens when faulty units are decommissioned or when extra units are deployed to increase throughput or fault tolerance. Another complication is *anonymity*, in the sense that cooperating robots may not necessarily rely on, or be aware of, each other's identity. For instance, the identity is irrelevant in a flock of drones that adjust their direction by looking at each other. Anonymity has a particularly disruptive impact on communication, as it renders existing traditional mechanisms such as point-to-point communication fundamentally inadequate. Another cause of concern with MRSs is *decentralization*, that is, the absence of a central entity to coordinate the robots' activities. Decentralization makes synchronization especially challenging, if possible at all, and in general makes it difficult to achieve an acceptable robustness of the interaction protocols. Another source of complexity in MRSs is their typically *large size*, in particular, the considerably large state space resulting from a large number of components asynchronously interacting with each other.

In fact, the above mentioned features are challenges to the specification of MRSs. A vast portion of literature relies on general-purpose languages to model MRSs (Pitonakova and Bullock, 2013; Buchanan et al., 2016; Pitonakova et al., 2016). But, typically, each work focuses on specific classes of systems and specific operating contexts. In general, modeling MRSs using general-purpose languages is not intuitive, and it can lead to increased code complexity and higher chances of introducing programming errors. Moreover, the resulting programs are hard to maintain and reasoning about them turns out to be complicated.

On the other hand, domain-specific languages with tailored and higher-level primitives can facilitate the specification of MRSs and the process of reasoning about them (Matarić, 1993). However, the heterogeneity of the domains might be detrimental for compositionality, and it might become harder to specify complex systems by composing available solutions. Adopting excessively specific formalisms is another risk, as the capability of the language to realistically describe scenarios of interest might be affected. Thus, linguistic support should intend to achieve an acceptable trade-off between expressiveness and generality.

The correctness and efficiency of an MRS is commonly assessed by means of simulations or testing in a possibly controlled environment. However, these techniques are not sufficient to predict complex collective behavior emerging from component interaction (Matarić, 1995). In particular, because of the large state space, a systematic exploration of all possible behaviors is often impractical, and subtle corner cases that lead to undesirable situations might be easily overlooked. Languages equipped with clear semantics can improve both informal reasoning and formal verification of properties, especially by exploiting higher-level information about the system to guide the analysis (Clarke et al., 1996; Flanagan and Godefroid, 2005). In the long term, such languages could become the core element of development platforms where automated reasoning tools can be seamlessly integrated.

In this paper, we review some languages and related development platforms stemming from MRS and multi-agent systems (MAS) literature. Our selection is driven by the different nature and goals of the chosen languages that are representative of wider categories of similar languages. Buzz (Pinciroli et al., 2015) is oriented to real-world applications and it shares some similarities with modern and popular languages, such as Python, JavaScript, and Lua; it focuses on simulation for system analysis. Interpreted Systems Programming Language (ISPL) and its surrounding framework (Lomuscio et al., 2009) are designed to support end to end development, and, specifically for automated reasoning by combining traditional analysis with advanced cutoff techniques. Finally, Software Component Ensemble Language (SCEL) (De Nicola et al., 2011) is a modular process description language where features of MRSs, such as anonymity and open-endedness, are naturally modeled, allowing a high degree of naturalness in the specification of individual behavior. In this research, we highlight the main characterizing features of these three formalisms and compare them by considering how they model several traits commonly found in MRSs. We also evaluate to what extent these languages can support the design phase through simulation or verification. We focus on languages based on *individual behavior design*. Thus, developers are expected to be mainly concerned with specifying the behavior of single components; the expected global behavior is not explicitly programmed, rather it is supposed to arise from the interaction of individuals and is a primary target for analysis. This bottom-up approach is still widely adopted because of its intuitiveness (Brambilla et al., 2013). Although alternative methods have been proposed, such as automatic design of individual behavior from higher-level specifications (Ulusoy et al., 2013; Francesca et al., 2015; Nikou et al., 2016; Nagavalli et al., 2017) or top-down behavioral languages (Bachrach et al., 2008), the lack of consistent empirical practices makes it hard to assess and compare these contributions (Francesca and Birattari, 2016).

To guide our comparison, we focus on two popular scenarios, namely, *foraging* and *flocking*. In foraging, robots search for items in the environment and take them to a fixed location. Flocking, on the other hand, is a process where robots that initially move in different directions eventually agree to head the same way. Both foraging and flocking are commonly observed in biological systems and are representative of most of the sources of complexity in MRSs.

This paper is structured as follows. In section 2, we describe a set of features commonly found in MRSs and elaborate as to how they represent a source of complexity during the specification or analysis of these systems. Section 3 introduces our selection of representative languages and compares them on their ability to model the aforementioned MRS features. In section 4, we describe our case studies and provide a basic implementation in each of the considered languages, along with observations made on their respective advantages and limitations. Section 5 briefly discusses other relevant languages and previous survey work in this area. We conclude with some final considerations and by mentioning possible research directions in section 6.

## 2. Common features of multi-robot systems

In this section, we identify some common features of MRSs. First of all, these systems may lack a central control unit; in other words, they might be *decentralized*. In this case, there is no reliable way for a component to obtain correct information about the global state of the system. Robots might also be free to join or leave the system at any time, a feature known as *open-endedness*. This may happen deliberately (e.g., robots returning to a home location to charge their batteries) or due to unexpected events, such as hardware failures. Open-endedness complicates reasoning; for instance, robots leaving the system create issues similar to those raised by failed processes in distributed computing (Lamport, 1978a). At the same time, robots joining a system might need to gather information from other components before being able to cooperate; as another example, a robot might have to find alternative solutions when a robot it was collaborating with on some task leaves the system. This calls for components with *self-managing* capabilities, such as self-configuration (Kephart and Chess, 2003). Furthermore, computational processes are *distributed*, both physically and logically. Physical distribution, among other things, exposes inter-process communication to significant delays and possible failures. On the other hand, logical displacement requires additional care to prevent the well-known risks of concurrent programming, such as deadlocks and process starvation. As a further source of complication, often there are no temporal constraints to local computation and interaction, as these systems may be partially or fully *asynchronous*. For instance, robots may take an arbitrarily long time to send or read a message, which can cause fundamental problems in distributed computing, such as the impossibility of distributed consensus (Fischer et al., 1985). Interaction in MRSs is also characterized by *anonymity*, in the sense that it typically does not rely on identity. Moreover, in a decentralized or open-ended system, the whole concept of identity is not easy to establish. The ability to select partners according to their current task, or their capabilities, can be more useful instead. For instance, robots in a foraging swarm can perform the task of recruiting by communicating the position of food to idle neighbors (Pitonakova et al., 2017). Such action only relies on the observed state of neighbors and, thus, can be performed even when the agents have no individual identity.

Owing to the abovementioned features, the common interaction patterns of concurrent and distributed systems, such as point-to-point communication, shared memory, or synchronization, turn out to be inadequate for MRSs. Therefore, different solutions have been proposed which are a better fit to large and open-ended systems that do not rely on the concept of identity. These include many-to-many communication (e.g., multicast or broadcast) or even group-oriented interaction. A group-oriented network is composed of groups of collaborating processes where messages target groups rather than individual processes (Birman, 1993). As a matter of fact, popular robots in the MRS literature, like the Kilobot (Rubenstein et al., 2012), even lack hardware tools for unicast or synchronous communication.

As the complexity of MRSs increases, there is a growing need for them to react to new environmental conditions without human support. This feature, known as *adaptivity*, is considered a necessity for future computing systems as a whole (Kephart and Chess, 2003), but it is especially attractive in the case of MRSs, since they are situated in a physical world where a large number of unexpected situations may arise. Adaptive behavior can be found in various biological systems such as ant colonies. When a source of food is found, ants collectively find an optimal path from the nest. If the path is disrupted, the colony is able to find a new and optimal path by relying on elementary actions performed by individual ants (Dorigo et al., 2006). This example also shows that system-wide adaptivity can be obtained from simple actions by individual components.

Moreover, robots can be different from one another: their behavior, equipment, and capabilities may be *heterogeneous*. In principle, it is always possible to describe a system with differentiated behavior as a homogeneous one, if the chosen formalism provides adequate control-flow statements. However, this approach can greatly increase the complexity of the resulting specifications. Therefore, very heterogeneous systems would quickly become hard to understand and maintain. Owing to the complex control-flow structure, tractability of automated reasoning would be affected too.

The *large size* of the system may hinder the feasibility of practical implementations, as it stresses on the underlying runtime environment and data structures. A large size is detrimental to verification and even simulation may become harder. These effects are further complicated by *nondeterminism and nonlinearity*. When the system is nondeterministic, multiple transitions are executable from a given state, complicating the analysis. Nonlinearity, on the other hand, means that a local transition may trigger a disproportionate system-wide effect. As a consequence, simulation not only ends up being more demanding but also less significant, as it might not spot critical yet subtle cases where the system fails. The size of a system also plays a role in the classification of MRSs. For instance, nearly-homogeneous MRSs with a very large number of components typically fall into the *swarm robotics* category. A quantitative definition classifies a system of size *N* as a swarm when 10^2^<*N* < < 10^23^, with the rationale that “Avogadro-large” systems are better treated with statistical tools (Beni, 2005; Hamann, 2018). Multi-robot systems represent a more generic classification as they can be smaller in size but more heterogeneous.

The presence of an *environment* plays a critical role in MRSs too. In fact, many applications of MRSs involve *sensing* and *actuation*, that is, gathering data from the external environment or manipulating it, respectively. Modeling these actions in the form of agent-to-agent interaction might be a feasible exercise under some formalisms; however, it would require additional guarantees of synchrony, atomicity, and consistency. A similar observation has also been made in the more general context of multi-agent systems (Weyns and Holvoet, 2004; Weyns et al., 2006). The manipulation of the environment can also work as a medium of indirect interaction between robots. This mechanism, known as *stigmergy*, is often found in biological systems (Grassé, 1959; Theraulaz and Bonabeau, 1999), and it has some benefits over direct message passing. For instance, it is inherently anonymous, as each agent simply reacts to changes in the environment without knowing who caused them. It is also considered a highly scalable solution (Ricci et al., 2006; Heylighen, 2016). While inaccuracies in sensing and actuation can lead to lossy information transfer, these advantages make stigmergic interaction attractive and to be widely studied (Arkin, 1992; Werfel et al., 2005; Pitonakova and Bullock, 2013).

Additional peculiarities of MRSs are related to the knowledge of robots. Each component has only *partial awareness* of the current system state and, possibly, even of its own state. For instance, a robot may be able to know the position of its neighbors but not that of robots that are farther away. In an open-ended system, a robot might not even know the size of the system itself. Even when robots are able to obtain information about the system, the said knowledge might be partial or outdated by the time it is accessed. This raises the problem of knowledge representation and propagation among components, which are essential to accomplish complex coordination tasks (Pitonakova et al., 2017). As any kind of shared memory is unacceptable in large and decentralized systems, resorting to distributed data structures may be the only feasible approach. However, the design of these structures must deal with the problems arising from the extremely dynamic nature of MRSs, which can lead to integrity and consistency problems.

## 3. Languages

In this section, we present a set of languages suitable for the specification and analysis of MRSs, namely, Buzz (Pinciroli et al., 2015), ISPL (Lomuscio et al., 2009), and SCEL (De Nicola et al., 2011). We then compare how well each language captures the MSR features identified in section 2. We chose these languages for their different nature and goals, which make them good representatives for the main existing categories of specification languages.

Buzz is oriented to real-world applications and provides a quite mature runtime environment, including a reference virtual machine and a simulation platform with an integrated physical engine. Its similarities with popular general-purpose languages, such as Python, JavaScript, and Lua, can also be considered an advantage. ISPL and its surrounding framework are specifically designed to support end-to-end development of MRSs. Appropriate primitives are provided to model the interaction among agents and of agents with the environment. Reasoning about the knowledge of (groups of) agents is formally supported by epistemic logics. Automated reasoning on the specifications is performed via traditional analysis and advanced cutoff techniques. Finally, SCEL is a process calculus that offers the possibility of naturally expressing features such as anonymity and open-endedness. This is made possible by its inherently group-oriented interaction primitives, which rely on dynamic ensembles formed by taking into account the exposed features of components. Thanks to its parametric semantics, the language can also be adapted to manage different knowledge models.

### 3.1. Buzz

Buzz (Pinciroli et al., 2015; Pinciroli and Beltrame, 2016) is a development environment for heterogeneous robot swarms. It is designed around a core language that provides a few communication and coordination primitives, and it can be extended to suit the needs of the user. As an example, Buzz supports asynchronous communication with neighbors: each component maintains a list of neighbors and can broadcast a key-value pair or listen (in a non-blocking fashion) for a given key. Swarms, that is, dynamic ensembles of robots, are a first-class abstraction in Buzz. Robots can join or leave an ensemble at runtime, and swarms can execute arbitrary functions. For example, one could design a system where an ensemble periodically broadcasts sensor data, while a second swarm receives these messages and uses them to take decisions.

A distinctive feature of Buzz is the virtual stigmergy. Stigmergies represent shared knowledge. A stigmergy is implemented in practice as a distributed key-value store, locally replicated for each robot, where entries propagate according to attached timestamps. In the case when two agents try to bind different values to the same stigmergy key, there is an initial phase where both entries spread across the system. However, if the swarm is connected (i.e., each robot has at least one neighbor and, thus, a chance to propagate its own knowledge), at some point the entry with the higher timestamp will be the only one left to propagate; therefore, it will eventually replace the entries in all the local replicas. The use of Lamport timestamps (Lamport, 1978b) avoids inconsistencies without resorting to a global clock. This mechanism gives some guarantees over the eventual consistency of all local copies of the virtual stigmergies.

Buzz is very marginally concerned with embodiment, that is, the fact that robots are distinct entities situated in and are able to interact with the physical world (Brooks, 1991). Indeed, any kind of sensing and actuation, including the robot's own movements, can only be modeled by extending the language with appropriate functions. This philosophy reduces the complexity of the core language, and it leaves the responsibility of correctly defining the intended semantics of any extension to the developer.

In Buzz, every robot has a unique identifier. Robot identifiers are attached to all messages and are used by the tie-breaking protocol of the virtual stigmergy. Moreover, the robots periodically broadcast their identifiers to refresh each other's lists of neighbors. This might raise scalability issues, especially in dense swarms where each robot has a large number of neighbors. Buzz also assumes that a robot, upon receiving a message, can automatically detect the position of the sender, thanks to *situated communication* equipment (Støy, 2001). This avoids the need for robots to declare their own position in the message payload, and it simplifies the maintenance of the neighbor lists. At the same time, situated communication devices currently face other limitations. For instance, message exchange is limited by the line of sight, which might be a problem in very cluttered environments. Some shortcomings of situated communication have also been overcome by relying on GPS coordinates or by using tracking systems in indoors scenarios.

Multi-robot systems programmed in Buzz can be simulated on the ARGoS platform (Pinciroli et al., 2012). The user sets up the simulation by specifying the number of robots, the Buzz script they execute, their equipment, and the spatial distribution of robots. A description of the arena, including any obstacles, is also part of the configuration. More recent work (St-Onge et al., 2017) shows that Buzz can be also integrated with the ROS middleware (Quigley et al., 2009) and the Gazebo simulation environment (Koenig and Howard, 2004). Even though the authors stress the fact that in Buzz all robots are supposed to execute the same script, we were actually able to use ARGoS to simulate a system where two groups of MarXbots (Bonani et al., 2010) execute slightly different scripts. We observed that robots could communicate with each other via a virtual stigmergy, even if they belong to different groups. However, we also noticed that different agents may have the same identifier. Even though our simulation behaved as expected, this violates the aforementioned assumption on the uniqueness of identifiers and, therefore, can lead to undesired consequences.

### 3.2. Interpreted systems programming language (ISPL)

ISPL (Lomuscio et al., 2009, 2017) is based on *interpreted systems*, a generalization of labeled transition systems where multiple labeled transition systems (LTSs) may synchronize on specific actions (Fagin et al., 1995). Each agent has a state (a set of user-defined variables) and a *protocol* that defines the actions it can perform given the current state. Changes to the local state are encoded in a *local evolution* function, which takes into account both the current local state and the actions performed by other agents. Communication is not explicit but rather obtained as an indirect result of such synchronized actions. The state of agents is encapsulated; agents can only observe each other's actions and eventually synchronize. The environment is an exception, as it is modeled by a distinct agent whose variables may be fully or partially observed by the other agents. Such a mechanism can capture different communication patterns involving an arbitrary number of agents, including the case of multiple agents simultaneously interacting with the environment.

This way of modeling interaction, while flexible, makes asynchronous interaction difficult to describe. Representing delayed message deliveries would require extra state variables and evolutions for each agent. Value-passing is difficult to describe as well. In principle, agents might encode each possible value as a different action. However, this would considerably increase the complexity of the specifications and still be hopeless in the case of infinite domains. ISPL does not cope well with anonymity and open-endedness, because it assumes a fixed system size, and its transitions explicitly take into account the actions of the agents.

Some of the above concerns have been addressed in the MCMAS-P framework (Kouvaros and Lomuscio, 2016b), where the size of the system is parameterized. The user specifies the behavior of each *kind* of agent in the system; in the verification phase, concrete systems are instantiated by creating a given number of agents for each kind.

In the MCMAS framework, verification is performed via model-checking of *epistemic properties*. An epistemic formalism is typically derived from an existing temporal logic by adding modalities to “reason about the knowledge of the agents in the system” (Lomuscio et al., 2017). Epistemic logic allows to naturally express properties such as “All agents eventually know φ” or “Agent *i* always knows φ,” where φ is another temporal or epistemic property. MCMAS originally supported the ATLK language, an extension of Alternating-time Temporal Logics (ATL) (Alur et al., 2002). A more recent implementation supports a significantly more expressive language, LDLK (Kong and Lomuscio, 2017). Thanks to cutoff techniques, verification of a property against a limited number of concrete systems can be sufficient to prove that all concrete systems derived from the same set of roles do satisfy the property. However, finding a cutoff for an arbitrary property is, in general, an undecidable problem. Hence, the cutoff search algorithm of MCMAS-P is sound but incomplete. Moreover, open-ended systems are still out of reach, as the size of each concrete instantiation is fixed anyway. MCMAS also supports interactive simulation, where the user can choose an initial state of the system and manually select a sequence of transitions to better understand the behavior of agents.

### 3.3. Software component ensemble language (SCEL)

SCEL (De Nicola et al., 2011, 2014b, 2015) is a formal language for the description and verification of collective adaptive systems (Hillston, 2014). In order to capture the highly dynamic nature of this class of systems, it naturally supports concepts such as open-endedness and anonymity. Communication in SCEL is deeply related to the concept of knowledge repositories. A knowledge repository is a container of knowledge items. The nature of repositories and items is not specified, in fact, the SCEL semantics is parametric with respect to their semantics. Besides the commonly-used tuple spaces (Gelernter, 1985), other kinds of repositories have been proposed. Among the others, soft constraint programming (Schiex et al., 1995) can be integrated into SCEL by defining repositories as constraint stores (Montanari et al., 2015). Each component is equipped with a set of *attributes*, which are named values exposed to the whole system. Attributes and values are stored in the knowledge repository of the component, and they can change throughout the evolution of the system. The set of exposed attributes, known as *interface*, can be dynamic as well.

Communication in SCEL is achieved by manipulating the knowledge repositories. In addition to inserting items (via the **put** action), a component can read or withdraw an item that matches a specified *pattern*, or template (via the **qry** and **get** actions, respectively). The operations can be either point-to-point or *attribute-based*, that is, involving only those components that satisfy a given predicate over their exposed attributes. This leads to a high degree of anonymity, as components do not need to know the identity of interaction partners nor to expose their own. Components can manipulate their own repository by using the special self identifier. The knowledge repository is also an abstraction layer for sensing and actuation. For instance, the intention of an agent to move toward a destination *d* could be represented by putting a (“*moveTo*”, *d*) item into its own repository. Another process can retrieve this tuple, drive the agent's motors accordingly, and potentially announce the result of the operation by inserting another tuple in the repository. Notice that the **put** action is the only non-blocking one. The blocking nature of **qry** and **get** is useful to implement various reactive patterns and to guarantee processes synchronization. Guards are naturally implemented by waiting until a specific item can be withdrawn from the component's own repository. When the semantic of repositories is similar to that of tuple spaces, *generative communication* patterns can be easily applied (Carriero and Gelernter, 1989; Carriero et al., 1994).

A weakness of SCEL is that implementing a runtime environment that fully respects its operational semantics is nontrivial. The jResp implementation (De Nicola et al., 2014b) provides three options in that respect. The first one relies on a centralized message broker, which may not be acceptable in a fully decentralized scenario. Alternatively, messages and predicates can be broadcasted over a bus-like network topology. In this case, receivers accept or reject a given message after evaluating the associated predicate over their own interface. This solution is completely decentralized, but evidently it does not scale well with the size of the system. The third option is a peer-to-peer topology based on the Scribe protocol (Castro et al., 2002).

With jResp, it is also possible to simulate the computational aspects of a SCEL system. However, physical simulation (such as the one offered in Buzz by the ARGoS simulator) is currently unavailable. Multiple verification approaches exist for systems specified in SCEL and its derivatives. The subset of SCEL without policies, known as SCELight, can be directly translated to Promela, thus allowing for model-checking through the SPIN tool (De Nicola et al., 2014a). The MISSCEL implementation (Belzner et al., 2014) enables simulation and logical model-checking within the Maude framework. Furthermore, MultiVeStA can be used to verify systems through statistical model-checking, a technique where the satisfaction of properties is checked against a finite number of executions. As the name suggests, this approach can only provide a statistical evidence that the required property is satisfied. At the same time, it is highly parallelizable and can provide insight on systems that are too large to be formally verified (Legay et al., 2010).

### 3.4. Comparison

We summarize in Table 1 our considerations about the three formalisms.

**Table 1:** Comparison of MRS support of the languages we considered.

	**Buzz**	**ISPL**	**SCEL**
*Open-endedness*	No	No	Yes
*Asynchrony*	Yes	No	Yes
*Anonymity*	Medium (neighbors)	Low	High
*Heterogeneity*	Low	High	High
*Communication*	Ranged broadcast	Multicast	Multicast
*Knowledge representation*	Local variables, virtual stigmergies	Local and environmental variables	Parametric (e.g., tuple spaces)
*Environment*	No	Yes	Yes (as an additional component)
*Semantics*	Reference implementation	Formal (Kripke structures)	Formal (SOS)
*Analysis*	Physics-based simulation (ARGoS, Gazebo)	Simulation, Model-checking (MCMAS)	Simulation (jResp); Model-checking (SPIN, Maude); statistical model checking (Vesta)

We found SCEL to be the only language capable of representing systems of dynamic size. SCEL's syntax, in fact, allows a component to “spawn” additional agents via the new keyword. Such a feature is crucial to specify fully open-ended MRSs in a natural way.

ISPL is different from both Buzz and SCEL in that it enables explicit specification of the environment. It is not clear whether implementing an environment on top of Buzz would be possible, as the language's primitives are fully asynchronous and oriented to concrete robots. Extending the language seems to be the most appropriate approach. Meanwhile, using one or more SCEL components to model an environment could be viable, since the language provides both asynchronous and synchronous mechanisms and a more flexible representation of knowledge. ISPL provides no primitives for asynchronous interaction nor value-passing. This means that replicating asynchronous features, while possible in principle, would be quite complex and might negatively affect verification efficiency. The different approaches to communication are also reflected in the support for anonymity. In ISPL, anonymity is low as interaction happens through synchronization with specific agents; the mediation of the environment can represent a solution, like in our flocking example below, but adds complexity to the specification. Buzz offers the possibility of broadcasting or receiving messages among neighbors, but its dependence on unique identifiers clashes with anonymity. In SCEL, by contrast, attribute-based actions are inherently anonymous, as senders and receivers do not need any information on their identities in order to communicate.

The three representative languages considered in this paper are quite different in terms of support for system analysis and verification. Buzz seems to be the only one equipped with a physics-based simulation environment. Such a functionality is particularly useful for understanding the possible agents' reactions in real-world conditions. On the other hand, Buzz's support for formal verification appears quite limited. For both ISPL and SCEL automated formal verification is instead a primary goal. Statistical model checking can help to reason about large MRSs, but to date, it has only been adopted in the context of SCEL systems. However, considering that Buzz provides a simulation environment and MCMAS can compute traces of a given ISPL system, support for statistical model checking seems relatively straightforward to add.

## 4. Case studies

### 4.1. Foraging

Foraging is considered a canonical case study in the literature related to MRS, and in particular to robotic swarms. It can model different kinds of scenarios, such as “waste retrieval” and “search and rescue” (Brambilla et al., 2013). Specifying a foraging MRS can show the capabilities of the chosen specification language with respect to different features of these systems, such as the representation of the agents' knowledge and their interaction with the environment through sensors and actuators.

**Buzz**. As Buzz lacks a notion of environment as well as synchronous communication primitives, food items have to be implemented as components, and *ad hoc* protocols must be set up to ensure consistency. The system consists of two *swarms*: food items and forager agents. Foragers perform a random walk and repeatedly broadcast a pickup request to all neighbors. Food items wait for pickup requests, and, if the requesting forager is close enough, they answer back with their identifier to indicate that they have successfully been collected.


**function**  food_listen(vid,  value,  rid)  {
  # Check **if** the robot is closer than 50 cm
  d  =  neighbors.get(rid).distance
  **if**  (d  <  50)  {
    neighbors.broadcast("response",  rid)
    #  Stop  responding  to  other  
foragers
    neighbors.ignore("pick_up")
  }
}
**function**  robot_listen(vid,  value,  rid) {
  **if**  (value  ==  id)  {
    log(id,  ":  picked  up  ",  rid)
  }
}
**function**  init()  {
  #  Robots  with  id  =  0,  4,  8  etc.  
are  food  items
  foodSwarm  =  swarm.create(1)
  foodSwarm.select(id  %  4  ==  0)
  #  All  the  others  are  foragers
  foragerSwarm  =  foodSwarm.others(2)
  **if**  (foodSwarm.in())  {
    neighbors.listen("pick_up",  food_listen)
  }  **else**  {
    neighbors.listen("response",  robot_listen)
  }
}
**function**  step()  {
  **if**  (foragerSwarm.in())  {
    #  Only  foragers  execute  this  block
    #  Random  walk  (sets  
linear/angular  velocity)
    gotop(5,  **math**.rng.uniform(-3.0,  3.0))
    neighbors.broadcast("pick_up",  id)
  }
}


We encode local behavior by implementing two standard Buzz functions. The first one is init(), which is executed only once at the moment of activating the robot. Function step(), instead, is repeatedly executed by the robot until the experiment terminates. One might also want food items to terminate once they are collected by foragers; however, the language appears to provide no facilities to block the execution of function step(). Another problem with our solution is that two foragers can pick up the same food item. Namely, if a food item receives two pick_up messages, it could perform the listener function food_listen twice and send two different response messages.

**ISPL**. Let us assume that the arena is a two-dimensional grid of size 10 × 10. We use the Environment agent to keep track of the position of food items and record whether they have been collected. We introduce two integer variables, food < i>X and food < i>Y, and one boolean variable food < i> for each food item *i*. We store them as *observable variables*, since, foraging robots need to access it. We also define an internal variable foundItems to count the number of collected items, which is needed for verification purposes. The robots, on the other hand, are agents with a position. We only give the specification for Robot1. The only difference between foragers is their identifier.


**Agent**  Robot1
  **Vars**:
    x  :  1  ..  10;
    y  :  1  ..  10;
  **end**  **Vars**
  -- ...
**end**  **Agent**


We define the protocol and evolution functions so that every forager performs a random walk and can pick up an item if both the forager and the item are exactly in the same position, and if the item has not previously been collected. When an item is collected, the environment updates the corresponding boolean variable and increments the counter.


-- *Environment*
**Evolution:**
  food1  =  **false**  **and**  foundItems  =  
foundItems+1  **if**
    (Robot1.Action  =  PickFood1);
-- *Repeat for all robots and food items*
**end  Evolution**
-- *Robots*
**Actions**  =  {Up,  Down,  Left,  Right,  
PickFood1,  PickFood2};
**Protocol:**
  y  <  10  :  {Up};
  y  >  1  :  {Down};
  x  <  10  :  {Right};
  x  >  1  :  {Left};
  Environment.food1  =  **true**  **and**
    x  =  Environment.food1X  **and**
    y  =  Environment.food1Y  :  
{PickFood1};
  -- *Repeat for all food items*
**end**  **Protocol**
**Evolution:**
  x  =  x+1  **if**  (**Action**  =  Right);
  x  =  x-1  **if**  (**Action**  =  Left);
  y  =  y+1  **if**  (**Action**  =  Up);
  y  =  y-1  **if**  (**Action**  =  Down);
**end**  **Evolution**


Finally, we define the initial state of the system, where all items are available and the foundItems counter is set to 0. Having specified no restriction on the position of the robots and the food items, all possible positions in the arena are nondeterministically considered.


**InitStates**
  Environment.food1  =  **true**  **and**
  Environment.food2  =  **true**  **and**
  Environment.foundItems  =  0;
**end**  **InitStates**


Alternatively, one could model food items as components. Parameterized versions of this approach are available in the literature (Kouvaros and Lomuscio, 2016b); however, these models do not seem to explicitly model the location of robots and items.

**SCEL**. Let us assume knowledge repositories to be tuple spaces. The implementation we describe is based on more complex examples, related to search and rescue operations, available in the existing literature on SCEL (De Nicola et al., 2014b, 2015). We model both foragers and food items as SCEL components. Each forager exposes its own position *pos*, the task it is performing (initially all robots are *idle*), and the range of its sensor. Each food item runs the same process *P*_*food*_; it repeatedly communicates its own position to all idle foragers within their sensors range, thus, allowing them to detect the item.

$$\begin{array}{l} P_{food}≜\;put\left(\text{"}food",this.pos\right)@(task="idle"\\ \wedge \|pos-this.pos\|\leq range).P_{food}\\ +\\ qry\left(\text{"}found"\right)@\;self.nil \end{array}$$

This process terminates when the item finds a (“*found”*) tuple in its own knowledge repository (note that nil denotes the inactive process). Before describing the behavior of foragers, let us assume that each food item initially has a (“*lock”*) tuple in its own repository. We use this tuple to make sure that only one forager at most is able to collect the item.

Foragers alternate between the idle and working states. In the idle state, they just perform a random walk until they sense a food item. In that case, they change their *task* attribute accordingly and move toward the food source.

$$\begin{array}{l} P_{idle}≜\;get\left(\text{"}food",?f\right)self.P_{work}\left(f\right)\\ \;+put\left(\text{"}randomWalk"\right)@\;self.P_{idle}\\ P_{work}\left(food\right)≜\;put\left(\text{"}task","work"\right)@\;self.\\ \;put\left(\text{"}moveTo","food"\right)@\;self.qry\left("reached","food"\right)\\ \;@\;self.(\\ \;get\left("lock"\right)@\;\left(pos=food\right).put\left("found"\right)\\ \;@\;\left(pos=food\right).\\ \;put\left(\text{"}task",\text{"}idle"\right)@\;self.P_{idle}\\ \;+\\ \;put\left("task",\text{"}idle"\right)@\;self.P_{idle}) \end{array}$$

The syntax (“*food*”, *?f*) denotes a template. In this case, the template is matched by all two-element tuples whose first element is “*food*”; when such a tuple is found, it is removed from the repository and its second element is bound to variable *f*. Similar to many other programming languages, *P*_*work*_(*f*) denotes a parametric invocation, whose actual parameter *f* is bound to the formal parameter *food*. As observed in section 3, we use the special tuple *(moveTo, p)* to indicate that the robot is moving toward position *p*. Similarly, we assume that a *reached* tuple is generated when the robot arrives at the destination specified in a previous *moveTo* tuple. Notice that updating the value of an attribute, such as *task*, needs no additional primitives, as it can be done by manipulating items in the local knowledge repository. To pick up an item, a robot first attempts withdrawing its *lock* tuple. When this is not possible, another forager would have already picked up the item. In this case, the robot simply goes back to the idle state.

### 4.2. Flocking

In flocking, agents start in a state of incoherent motion, but they eventually agree to move in the same direction. This is an example of emerging behavior and a basic instance of a consensus problem, which is fundamental for many cooperative tasks (Valentini et al., 2017).

Excluding those cases where the language provides more appropriate primitives, in this section, we program this behavior by specifying a variation of the *voter model*. In a voter model, agents (voters) are seen as nodes of a graph. Each one of them has an initial *opinion*, picked from a finite number of options (Liggett, 2005). During the evolution of the system, an agent can observe and copy the opinion of any of his neighbors. A voter model can either converge (i.e., all voters eventually adopt the same opinion) or oscillate indefinitely. This depends on factors such as the initial distribution of opinions and the topology of the graph.

**Buzz**. A virtual stigmergy is particularly intuitive to model this behavior, making sure that the swarm eventually agrees on a direction.


**function**  init()  {
    **math**.rng.setseed(id*id)
    my_yaw  =  **math**.rng.uniform(0,  2.0  *  
**math**.pi)
    vstig  =  **stigmergy**.create(1)
    vstig.put("yaw",  my_yaw)
}
**function**  step()  {
    **var**  cur_yaw  =  **pose**.orientation.yaw  
%  (2.  *  **math**.pi)
    **var**  err  =  vstig.get("yaw")  -  
cur_yaw
    control(err)
}


In the init() function we use the (squared) identifier of the agent as a seed for the pseudorandom number generator. This operation is required to make agents behave differently during simulations. We then generate a random value for the direction and put it into a virtual stigmergy.

At each execution step, a robot retrieves the direction from the stigmergy by calling the vstig.get() function and computes the current error (i.e., the difference between the desired yaw angle and the current one). The control() function then rotates the robot if the error is too big, otherwise moves it forward.

Every time a Buzz robot reads a value from the stigmergy, it automatically sends that value to its neighbors, asking them to confirm whether it is up-to-date. Neighbors in turn use this information to either update their own local copies or to reply with a more recent value if they have one. This interaction pattern makes it easier for all robots to converge to a common value, even when parts of the swarm become temporarily disconnected from the rest.

A basic voter model can also be described in Buzz. We can use the communication primitives of the language, so that each robot repeatedly broadcasts its opinion among its neighbors. Periodically, robots listen to these messages and adjust their direction accordingly.


t  =  0
 
**function**  init()  {
    **math**.rng.setseed(id*id)
    my_yaw  =  **math**.rng.uniform(0,2)  *  
**math**.pi
    **neighbors**.listen("yaw",  
**function**(vid,  value,  rid)  {
       **if**(value  !=  my_yaw  **and**  (t  %  
20)  ==  0)  {
           my_yaw  =  value
           log(id,  "  change  to  ", 
           my_yaw)
       }
    })
}
**function**  step()  {
    t  =  t  +  1
    **var**  cur_yaw  =  **pose**.orientation.yaw  
%  (2.  *  **math**.pi)
    **var**  err  =  my_yaw  -  cur_yaw
    control(err)
    **neighbors**.broadcast("yaw",  my_yaw)
}


Here the condition (t % 20) == 0 means that robots can only attempt to change their opinion once every 20 time steps. By altering this expression it is possible to capture interesting variations, for example, where the waiting time of each robot follows an exponential (Cox, 1989) or power-law (Takaguchi and Masuda, 2011) distribution; the behavior of *zealots*, that is, agents that never change their opinion (Mobilia et al., 2007), can also be specified in this way.

**ISPL**. We decided not to rely on explicit communication between robots. Doing so would, in fact, require appropriate protocol and evolution rules for each pair of agents. The size of the specifications would, therefore, grow quadratically with the number of robots. Our implementation, again, takes advantage of the Environment agent. Each robot starts with an arbitrary direction in its state. At any moment, a robot can move in its stored direction, or it can watch and imitate the direction of the robot that moved last.


**Agent**  Environment
  **Obsvars**:
    dir  :  {Up,  Down,  Left,  Right};
  **end**  **Obsvars**
  -- ...
  **Evolution**:
    dir  =  Up  **if**  (Robot1.Action  =  Up);

    -- *Repeat for all robots and directions*
  **end**  **Evolution**
**end**  **Agent**
**Agent**  Robot1
  **Vars:**
    x  :  1  ..  10;
    y  :  1  ..  10;
    dir  :  {Up,  Down,  Left,  Right};
  **end**  **Vars**
  **Actions**  =  {Up,  Down,  Left,  Right,  
Watch};
  **Protocol**:
    dir  =  Up  **and**  y  <  10:  {Up,  
Watch};
    -- *Similarly for other directions*
    Other:  {Watch};
  **end**  **Protocol**
  **Evolution**:
    dir  =  Environment.dir  **if**  (Action  
=  Watch);
    y  =  y+1  **if**  (Action  =  Up);
    -- *Similarly for other directions*
  **end**  **Evolution**
**end**  **Agent**


We define the protocol so that robots cannot move in their chosen direction if they are on the edge of the arena. For instance, an agent at position (3, 10) cannot go Up and is only allowed to perform the Watch action. We encode this behavior through the special condition Other, which holds in all the states that do not match the other protocol rules. We used the MCMAS model checker to verify our implementation against a property of the form **AF** *consensus* (for all possible executions, eventually, the *consensus* proposition will hold). The model checker successfully proved that a team of two robots will always reach a state where they move in the same direction. However, in the presence of three or more robots, we were able to find cyclic traces where consensus is never achieved.

We can slightly alter the description of robots, so that they can move across an edge and get to the opposite side of the grid. For instance, a robot at (1, 4) will be able to move left and reach (10, 4). In other words, we consider a toroidal arena rather than a square one. Introducing this change makes consensus unreachable, even with just two robots.


**Protocol:**
  dir  =  Up  :  {Up,  Watch};
  -- ...
**end**  **Protocol**
**Evolution:**
  y  =  1  **if**  (Action  =  Up  **and**  y  =  
10);
  y  =  y+1  **if**  (Action  =  Up  **and**  y  <  
10);
  -- ...
**end  Evolution**


While ISPL cannot express voter models in a natural way, a dialect of the language (ISPL-OFP) has been proposed to model and verify an array of opinion formation protocols (Kouvaros and Lomuscio, 2016a).

**SCEL**. Modeling a basic voter model in SCEL is simple. Each component exposes its current position and direction, and it uses the **qry** action to copy the direction of a neighbor. Owing to the semantics of the action, a neighbor is selected nondeterministically among the components that satisfy an attribute-based predicate. In this example, the predicate targets robots with a different direction and within a given communication range.

$$\begin{array}{l} P≜\;qry\left("direction",?d\right)@(\|pos-self.pos\|\leq self.range\\ \;\wedge \;direction\neq self.direction).\\ put\left("direction",d\right)@self.P \end{array}$$

We can further refine this behavior by introducing logical clocks (Lamport, 1978b), obtaining a similar protocol to the one used in Buzz for virtual stigmergies. Let us suppose that each component has a (“*time*”, 0) tuple. This value is also exposed as the *time* attribute of the component. We refer to this attribute in the **qry** predicates to ignore out-of-date neighbors. We also attach a timestamp to *direction* tuples. Whenever a component finds a neighbor with a timestamp *t* higher than its own, it sets its clock to *t*+1. With a similar approach, robots can divide the computation into “rounds” and solve more complex problems, such as distributed graph coloring (see e.g., in Abd Alrahman et al., 2017).

$$\begin{array}{l} P≜\;qry\left("direction",?d,?t\right)@(\|pos-self.pos\|\leq self.range\\ \;\wedge \;time\neq self.time).\\ \;put\left("time",t+1\right)@self.\\ \;put\left("direction",d,t+1\right)@self.P \end{array}$$

## 5. Other work

**Languages and Frameworks**. The PARS process algebra (Steenstrup et al., 1983) follows an approach similar to the one of ISPL (section 3.2), with formal semantics given in terms of port automata. The primitives for communication are synchronous, but a specific operator allows to additionally model asynchronous message passing with explicit timing. This line of work mostly focuses on guaranteeing safety of emergent behavior (Lyons and Arkin, 2004). Similarly, the P language (Desai et al., 2013; Desai and Qadeer, 2017) allows to define a collection of state machines interacting through point-to-point and asynchronous messaging. Both PARS and P model the environment as a separate automaton and have been used as part of integrated verification frameworks for MRSs (Lyons et al., 2014; Desai et al., 2017). In MASL, communication is obtained by remote procedure calls that may be either synchronous or asynchronous, thanks to synchronization constructs known as futures (Baker and Hewitt, 1977). Interaction can also happen via shared variables. The language provides mechanisms to restrict the execution of a block of code to a subset of agents (similar to the filtering primitives in Buzz) or to enforce synchronization in groups of agents (Dubois et al., 2009). The popular ROS middleware (Quigley et al., 2009) provides libraries for existing general-purpose languages such as C++ and Python, and it supports asynchronous message passing based on the publish-subscribe pattern (Birman and Joseph, 1987) as well as synchronous calls to remote procedures (known as *services*).

Another viable approach to multi-robot programming is concurrent logic programming. The basic elements of logic programming languages naturally correspond to the common traits of MRSs. For instance, input unification, guarded clauses, and process reduction can be used to obtain synchronization, to model self-awareness, and to partition a goal into subtasks, respectively (Ben-Arieh and Maimon, 1991). Along this line of research, further programming constructs have more recently been proposed to improve the specification of MRSs, especially, for expressing the physical capabilities of robots and for introducing explicit timing constraints (Jiang et al., 2016).

Another family of languages is based on the belief-desire-intention (BDI) paradigm (Rao and Georgeff, 1995). Agents perform *plans* in order to fulfil some *goal* or desire. Local knowledge is represented by a set of *beliefs*. Interaction is typically modeled by adding or removing beliefs. For instance, 3APL (Hindriks et al., 1999; Dastani et al., 2003) offers a point-to-point communication primitive to synchronously update the belief base of both the receiver and the sender, which maintains the sent messages as a set of beliefs. The Jason language (Bordini et al., 2007) extends this mechanism to asynchronous and multicast actions, allowing to exchange either beliefs or plans (similar to process exchange in SCEL). The JaCaMo framework (Boissier et al., 2013) combines Jason with the CArtAgO language for environment modeling. On the other hand, ALICA (Skubch et al., 2011; Skubch, 2013) does not provide explicit message passing. Any information relevant to task planning, such as confirming the successful execution of a plan or requesting synchronized actions, is automatically broadcasted. Based on this information exchange, agents can infer and act upon the state of other agents. ALICA has more recently been extended (Saur and Geihs, 2015) to support integration with the pRoPhEt planning engine for MASs (Saur and Geihs, 2014).

**Related Reviews**. Several surveys of robotics languages and development platforms have been proposed (Kramer and Scheutz, 2007; Chitic et al., 2014; Nordmann et al., 2014), but none of them address the typical sources of complexity of MRSs discussed in this paper. The literature is also rich in taxonomies for specific aspects found in MRSs, such as coordination (Yan et al., 2013) and task allocation (Gerkey and Matarić, 2004).

A variety of language surveys can be found in the broader context of multi-agent systems (Mascardi et al., 2005; Bordini et al., 2006; Feraud and Galland, 2017). However, those surveys do not include a comparison of the languages in terms of the relationship between their features and the key features of MRSs that we have highlighted in this paper. Attempts have been made, based on the study that focuses on the relation between the simple local behavior and the complex emergent behavior occurring in natural systems, to draw some general principles about the architecture of MASs (Parunak, 1997). The peculiar traits of complex systems in general, such as emergence, have been the subject of a substantial amount of research (Heylighen, 1989; Barabasi and Albert, 1999; Odell, 2002).

A number of mechanisms for information exchange in foraging, that are a fundamental and highly reusable element of MRSs, have been proposed in Pitonakova et al. (2018). The need of distinguish between individual knowledge and external repositories of information, such as the environment, has also been stressed in Pitonakova et al. (2017).

## 6. Conclusions and future work

In this work, we have identified a number of features typically found in multi-robot systems. These traits can make MRSs hard to design, implement, and reason about. We have presented a selection of languages, showing how specialized linguistic primitives can help in the specification of such systems. We have also compared the languages in terms of support for automated analysis. To better understand the strengths and weaknesses of each language, we considered two popular case studies from the MRS literature and provided basic implementation in the surveyed languages. We used these implementations to show how specific abstractions provided by the languages facilitates modeling nontrivial behavior. For instance, the presence of synchronous operations is vital for scenarios where the interaction with the environment is predominant. At the same time, we have seen that group-oriented forms of interaction, either among neighbors or based on the more general framework of attribute-based communication, allow for a more natural specification of nontrivial cooperation patterns. Our work is by no means an exhaustive review of the state-of-the-art research. We focused on choosing a representative set of languages to allow an effective qualitative comparison of existing languages. Additional languages and frameworks, such as those briefly introduced in Section 5, could be the subject of further investigation. Describing and clarifying the factors that make MRSs distinctively challenging is also an important first step toward the specification of new MRS-oriented languages, which is another possible direction of research.

**Future Work**. This work is, for us, instrumental to design a new language for multi-agent systems by building on the lesson learned from the three languages we have surveyed. The language we are aiming at should make an intuitive design of local specifications and automated analysis of global properties and emerging behaviors possible. We intend to design a language that combines the stigmergic interaction of Buzz with the attribute-based communication of SCEL, whose agents will interact by manipulating and asynchronously propagating their limited share of knowledge. The language will be equipped with formal semantics to enable automated verification of logical properties by building on tools and methods developed for ISPL.

## Author contributions

RD, LD and OI contributed to the introduction, features of MRSs, related and future work. LD and OI implemented the surveyed case studies with discussions. LD contributed to the definition of languages and case studies of interest, language comparison and conclusions.

### Conflict of interest statement

The authors declare that the research was conducted in the absence of any commercial or financial relationships that could be construed as a potential conflict of interest.
